# Prescription Pattern and Its Influencing Factors in Chinese County Hospitals: A Retrospective Cross-Sectional Study

**DOI:** 10.1371/journal.pone.0063225

**Published:** 2013-05-10

**Authors:** Heng Wang, NianNian Li, Haidi Zhu, Shuman Xu, Hua Lu, ZhanChun Feng

**Affiliations:** 1 HuaZhong University of Science and Technology, Wuhan, Hubei, China; 2 The First Affiliated Hospital of Anhui Medical University, Hefei, Anhui, China; 3 Social Science and Health Management School, Anhui Medical University, Hefei, Anhui, China; University of Sao Paulo, Brazil

## Abstract

**Objective:**

This study aimed to investigate prescription patterns and influencing factors in Chinese county hospitals.

**Methods:**

Prescription quality was evaluated by five indicators proposed by WHO/INRUD. A questionnaire for doctors was designed by our research group. All internists, surgeons, obstetricians, gynecologists and pediatricians from 10 county hospitals in Anhui province were asked to fill the questionnaire. Their prescriptions from May 2011 to April 2012 were analyzed.

**Results:**

Three-hundred and thirty-seven doctors completed valid questionnaires, and 5099 prescriptions were analyzed. The average number of drugs per prescription was 3.52±2.31; the average percentage of generic drugs, antibiotic usage, injection drug usage, and drugs prescribed from the national essential drug list were 96.12%, 29.90%, 20.02% and 48.85%, respectively. Differences in final academic degree and specialty led to differences in all of the five prescription quality indicators. The older doctors tended to use more antibiotics. Doctors with more education, more training on rational drug use, and better acquisition of medicine knowledge prescribe a lower percentage of generic drugs. Moreover, the more supportive the doctor’s attitude to national essential medicine policy, the higher the percentage of generic drugs were prescribed. A higher level of medical knowledge was associated with a higher percentage of drugs prescribed from the essential drugs list.

**Conclusions:**

Promoting the education of medical knowledge on doctors, reinforcing the publicity of rational drug use to doctors, and initiating the performance evaluation for doctors are effective ways for improving prescription quality in Chinese county hospitals.

## Introduction

It is estimated by the World Health Organization (WHO) that approximately 5.3 trillion U.S. dollars were spent on health services, and 25% of this amount was used to pay for medicines (from the WHO National Health Accounts data files for 2006) [1], [2]. However, almost half of the world’s population cannot afford these drugs. Data from the WHO indicates that by 2015, over 10 million deaths per year could be avoided by effective health interventions, such as national essential medicine policies [3].

“Essential medicines”, a concept proposed by WHO in 1977, was defined as drugs with availability, safety, effectiveness and rational use [4]. The WHO guideline on essential medicines has obtained approval from its member states. In 2009, the Chinese government approved the national essential medicines system. It is common sense that doctors with prescription eligibility should be responsible for rational drug use. An inappropriate prescribing pattern by doctors often encourages inappropriate self-medication by patients because of the asymmetry of medical information [5]. Doctors’ prescription patterns are not static or standardized but dynamic and individual [6]. Thus, studies on medicines are complicated social themes rather than medical themes [7].

To increase prescription quality and improve the rationality of drug use, we need to investigate the subjective and objective factors that affect doctors’ prescription patterns. Previous studies have shown that factors such as gender, age, educational status, specialty, work experience, and economic stimulation influenced their prescribing pattern. However, there were limitations in existing studies: all of the existing studies were single factor analyses, which may have ignored the confounding effects on doctors’ prescription patterns. Moreover, there was no study concerning the corresponding relationship between a doctor’s condition and his or her prescription records. In the present study, we integrated surveys on doctors with their prescription records to reveal subjective and objective factors that influence doctors’ prescription quality in Anhui province, China.

## Methods

### Ethics Statement

All of the research methods and investigational tools in this study were approved by the Ethics Committee of the First Affiliated Hospital of Anhui Medical University. All of the respondents in this manuscript gave a written informed consent to participate in the study, provided consent before filling out the questionnaire, and consented to the publication of the data.

### Study Design

A retrospective cross-sectional study was used to analyze the relationship between influencing factors and prescription quality indicators. Questionnaires for doctors were designed based on a literature review and expert consultation, and completed by the doctors themselves in county hospitals. Meanwhile, we selected some of the prescription records in the previous year for those who completed the questionnaires and filled out the Prescription Quality Indicator Statistical Table, which was designed to summarize the five prescription indicators for the following analysis.

### Study Tool

Five prescribing indicators proposed by WHO/INRUD (International Network for the Rational Use of Drugs) were used as the prescription quality evaluation tool in this study. These indicators included the average number of drugs per prescription, the percentage of drugs prescribed by generic name, the percentage of antibiotics per prescription, the percentage of injections per prescription, and the percentage of drugs prescribed from China’s national essential drugs list (2011). Averages of the above five indicators were calculated and integrated into the questionnaire for doctors.

Several possible influencing factors on doctors’ prescription quality were summarized by means of literature review, including their general conditions (age, gender, final academic degree, work experience, daily outpatient number and specialty), education and training experience on rational drug use, acquisition of medical knowledge, attitude toward the Chinese essential medicine policy and patient-related factors. The questionnaire for interviewing doctors was designed in accordance with the above factors and a pilot investigation was undertaken to test its reliability and construct validity. Except for the part of doctor’s general conditions, six common factors were extracted from the questionnaire, which are the education and training experience on rational drug use, acquisition of medical knowledge, attitude toward the Chinese essential medicine policy, job satisfaction, patients’ demographic characteristics and patients’ financial situation.

### Sampling Method

Anhui province is located in the central and eastern China, which has a population of 62.199 million. Hefei, Wuhu and Ma Anshan are three typical cities of this province. Ten county hospitals in the three cities were selected for investigation. Cluster sampling was used to investigate doctors in these hospitals. Rosters of all internists, surgeons, obstetricians, gynecologists and pediatricians were obtained from the medical administration departments, and the doctors’ names were numbered in order. Questionnaires with a doctor identification number were handed out to corresponding doctors to fill in. There were a total of 372 doctors with prescription eligibility, excluding those who were not on duty on the survey period, and 337 doctors completed the questionnaires. For surgeons, obstetricians, gynecologists and pediatricians who filled out the questionnaire, the first prescriptions in every month from May 2011 to April 2012 were written in the Prescription Quality Indicator Statistical Table by trained postgraduate students. Because internists have more subspecialties and use medicine as the main therapeutic method, we selected their first prescription every half-month during the same period.

### Analysis Method

All collected data were entered into EpiData 3.0 twice to ensure accuracy. Statistical analysis was conducted using SPSS (version 16.0). Single factor analysis of influencing factors on prescribing quality was performed by means of a Kruskal-Wallis test and correlation analysis. Multiple linear regression was used to conduct the multivariate analysis. The level of significance was set at P≤0.05.

## Results

Total of 372 doctors were eligible for this study, 337 valid questionnaires were obtained, and the valid response rate was 90.6%. A total of 5460 prescription records were selected and recorded in the Prescription Quality Indicator Statistical Table, of which 361 records were rejected due to incomplete information. Finally, 5099 (93.4%) valid prescriptions were included in the analysis.

### General Conditions of Doctors and Overall Prescription Quality

Age, gender, final academic degree, work experience, daily number of patients and specialty distribution are shown in Table 1.

**Table 1:** General conditions of 337 doctors.

Variable	Group	Number	Percentage (%)
**Age**	≤35 years old	150	44.5
	36–45 years old	127	37.7
	≥46 years old*	60	17.8
**Gender**	Male*	194	57.6
	Female	143	42.4
**Final academic degree**	Associate’s degree	35	10.4
	Bachelor’s degree	275	81.6
	Postgraduate and above*	27	8.0
**Work experience**	<10 years	129	38.3
	10–14 years	65	19.3
	15–19 years	52	15.4
	20–24 years	43	12.8
	≥25 years*	48	14.2
**Daily outpatient number**	<15	122	36.2
	15–50	171	50.7
	≥51*	44	14.2
**Specialty**	Internist	118	35.0
	Surgeon	67	19.9
	Obstetrician & gynecologist	91	27.0
	Pediatrician*	61	18.1

* Control groups of dummy variable setting of independent variables in multiple linear regression analysis.

As for the quality indicators of the 5099 prescriptions, the minimum number of drugs per prescription was 1 and the maximum was 10, with an average of 3.52±2.31; the average percentage of generic drugs prescribed was 96.12% (26%–100%); the average percentage of antibiotics usage was 29.90% (0%–100%); the average percentage of injection usage was 20.02% (0%–100%), and the average percentage of drugs prescribed from the national essential drug list was 48.85%(0%–100%).

### Single Factor Analysis of the Elements that Affected Prescription Quality

The six categorical variables (age, gender, final academic degree, work experience, average daily outpatient number and specialty) were regarded as independent variables. Meanwhile, the five indicators of prescription quality were regarded as dependent variables. Chi-square test was used because the five indicators were in non-normal distribution. The differences in gender, final academic degree and specialty led to the distinctions of average number of drugs per prescription. The variance of the utilization rate of generic names was caused by differences in age, gender, final academic degree, work experience and specialty. The differences in age, final academic degree and specialty led to the diversity of the percentage of antibiotics usage. The differences in age, gender and specialty led to the diversity in the percentage of injection usage. The percentage of drugs prescribed from the national essential drug list varied along with the differences in age, gender, final academic degree, work experience, average daily outpatient number and specialty (Table 2).

**Table 2:** Single factor non-parametric test results of prescription quality indicators.

Variable		Drug number	Percentage of generics	Percentage of antibiotics	Percentage of injections	Percentage of essential drugs
**Age**	≤35 years old	3.628	0.973	0.302	0.229	0.456
	36–45 years old	3.455	0.957	0.272	0.185	0.527
	≥46 years old	3.425	0.943	0.350	0.162	0.489
	X^2^	0.038	7.492	9.282	6.244	8.429
	p	0.981	**0.024** *	**0.010** *	**0.044** *	**0.015** *
**Gender**	Male	3.308	0.944	0.298	0.181	0.535
	Female	3.823	0.985	0.301	0.226	0.426
	X^2^	7.513	11.786	0.247	5.573	21.558
	p	**0.006** *	**0.001** *	0.619	**0.018** *	**0.000** *
**Final academic degree**	Associate’s degree	3.621	0.929	0.275	0.214	0.545
	Bachelor’s degree	3.393	0.962	0.310	0.193	0.490
	Postgraduate and above	4.760	0.996	0.215	0.260	0.401
	X^2^	7.970	8.298	11.122	5.741	12.029
	p	**0.019** *	**0.016** *	**0.004** *	0.057	**0.002** *
**Work experience**	<10 years	3.537	0.969	0.309	0.222	0.449
	10–14 years	3.700	0.975	0.274	0.215	0.503
	15–19 years	3.331	0.950	0.271	0.180	0.526
	20–24 years	3.306	0.953	0.299	0.168	0.542
	≥25 years	3.674	.0941	0.336	0.172	0.487
	X^2^	1.647	9.794	4.624	4.840	10.661
	p	0.800	**0.044** *	0.328	0.304	**0.031** *
**Daily outpatient number**	<15	3.459	0.972	0.2877	0.221	0.494
	15–50	3.628	0.950	0.2996	0.187	0.513
	≥51	3.320	0.977	0.3277	0.194	0.379
	X^2^	2.358	4.636	3.304	0.843	12.844
	p	0.308	0.098	0.192	0.656	**0.002** *
**Specialty**	Internist	3.085	0.957	0.281	0.184	0.515
	Surgeon	3.399	0.916	0.324	0.153	0.566
	Obstetrician &gynecologist	3.986	0.991	0.329	0.231	0.398
	Pediatrician	3.842	0.975	0.261	0.237	0.488
	X^2^	21.907	23.587	8.239	12.763	23.895
	p	**0.000** *	**0.000** *	**0.041** *	**0.005** *	**0.000** *

* Significance.

Single factor linear correlation analysis was performed between the five prescription quality indicators and the six principal components extracted from the questionnaires for doctors (acquisition of pharmaceutical knowledge, job satisfaction, education and training experience of rational drug use, attitude towards policy of essential medicines, patient’s demographic characteristics and patient’s financial situation). Different education and training experience resulted in the differences in the percentage of generic drugs prescribed. The variance in the attitude towards the essential medicine policy could elicit the diversity of the percentage of antibiotic usage. The diversity of patients’ demographic characteristics and financial situation would lead to the differences in the percentage of drugs prescribed from the national essential drug list. All differences stated above were significant (Table 3).

**Table 3:** Results of single factor linear correlation analysis prescription quality indicators.

		Drug number	Percentage of generics	Percentage of antibiotics	Percentage of injections	Percentage of essential drugs
**Education& training**	**r(p)**	0.006(0.907)	**−0.084(0.001** * **)**	0.010(0.857)	0.0391(0.478)	0.102(0.061)
**Acquisition of pharmaceutical knowledge**	**r(p)**	**−**0.053(0.333)	**−**0.100(0.066)	0.001(0.987)	**−**0.064(0.241)	0.099(0.071)
**Attitude toward Chinese essential medicine policy**	**r(p)**	**−**0.096(0.080)	0.044(0.420)	**−**0.010(0.849)	**−**0.062 (0.258)	0.059(0.279)
**Job satisfaction**	**r(p)**	**−**0.003(0.962)	**−**0.006(0.916)	0.057(0.297)	**−**0.068(0.216)	0.049(0.370)
**Patient demographics**	**r(p)**	0.010(0.859)	**−**0.070 (0.200)	0.000(0.995)	0.083(0.127)	0.094(0.084)
**Patient financial situation**	**r(p)**	**−**0.046(0.404)	**−**0.076(0.164)	0.002(0.964)	**−**0.033(0.547)	**0.114(0.037** * **)**

* Significant.

### Multifactor Analysis of the Influencing Factors of Prescription Quality

In present study, we used multiple linear regressions to analyze the relationship between the influencing factors and prescription quality indicators. The five indicators of prescription quality were taken as dependent variables. Eleven variables (age, gender, final academic degree, work experience, average daily outpatient number, specialty, education and training experience of rational drug use, acquisition of pharmaceutical knowledge, attitude towards essential medicines policy, patients’ demographic characteristics and patients’ financial situation) that were significant in the single factor analysis were used as independent variables. The forward method was applied to introduce the variables into the regression equation, with the inclusion criteria α equal to 0.05 and the exclusion criteria α up to 0.1. First, dummy variables for the five multi-categorical variables (age, final academic degree, work experience, average daily outpatient number, and specialty) were set. Then, the dummy variables of each dimension were introduced into the model as a whole and brought into the equation. The study showed that there was no relationship between age, gender, job satisfaction and patients’ financial situation with the five prescription quality indicators.

The average number of drugs prescribed by doctors with associate or bachelor’s degrees was significantly lower than those who had master’s degree or above. Meanwhile, the average drug number per prescription of the internists was lower than that of the pediatricians (see Table 4). Table 5 showed that the percentage of generic drugs prescribed by doctors with associate’s degrees was lower than doctors with a master’s degree or above. The percentage of generic drugs prescribed by surgeons was lower than that of pediatricians. With an increase in education and training, or an improvement in acquisition of pharmaceutical knowledge, the percentage of generic drugs prescribed slightly declined. The more positive an attitude toward the national essential drug policy by the doctor, the higher the percentage of generic drugs prescribed.

**Table 4:** Results of multiple linear regression analysis influencing factors on average drug number per prescription.

		Unstandardized coefficients		Standardized coefficients	t	P.
	Variable	B	Std. error	Beta		
	**(Constant)**	5.465	.742		7.370	.000
**Age**	**≤35 years old**	.897	.862	.193	1.041	.299
	**36–45 years old**	.383	.689	.080	.556	.579
**Gender**	**Female**	.135	.384	.029	.351	.726
**Final academic** **degree**	**Associate’s degree**	**−**1.854	.639	−.245	**−**2.902	**.004** *
	**Bachelor’s degree**	**−**1.954	.489	−.328	**−**3.994	**.000** *
**Work experience**	**<10 years**	**−**1.158	.941	−244	**−**1.231	.219
	**10–14 years**	−.668	.826	−.114	−.809	.419
	**15–19 years**	−.920	.805	−.144	**−**1.143	.254
	**20–24 years**	−.572	.654	−.083	−.876	.382
**Daily outpatient** **number**	**<15**	.382	.434	.080	.880	.379
	**15–50**	.554	.390	.120	1.422	.156
**Specialty**	**Internist**	**−**1.018	.379	−.211	**−**2.683	.008*
	**Surgeon**	−.596	.431	−.103	**−**1.382	.168
	**Obstetrician &** **gynecologist**	.261	.438	.050	.595	.552

* Significant.

**Table 5:** Results of multiple linear regression analysis influencing factors on average percentage of drugs prescribed by generic name.

		Unstandardized coefficients		Standardized coefficients	t	P.
	Variable	B	Std. Error	Beta		
	**(Constant)**	1.073	.043		25.156	.000
**Age**	**≤35 years old**	−.007	.038	−.034	−.191	.849
	**36–45 years old**	−.013	.031	−.059	−.422	.673
**Gender**	**Female**	−.008	.017	−.038	−.476	.635
**Final academic degree**	**Associate’s degree**	−.062	.028	−.179	**−**2.180	**.030** *
	**Bachelor’s degree**	−.038	.022	−.141	**−**1.747	.082
**Work experience**	**<10 years**	.009	.042	.041	.213	.832
	**10–14 years**	.027	.037	.102	.748	.455
	**15–19 years**	.012	.036	.039	.323	.747
	**20–24 years**	.011	.029	.036	.388	.698
**Daily outpatient** **number**	**<15**	.019	.019	.086	.971	.332
	**15–50**	−.010	.017	−.047	−.569	.570
**Specialty**	**Internist**	−.028	.017	−.127	**−**1.676	.095
	**Surgeon**	−.054	.019	−.204	**−**2.813	.005
	**Obstetrician& gynecologist**	.017	.020	.072	.860	.391
**Education& training**		−.006	.002	−.182	**−**3.326	**.001** *
**Attitude toward Chinese essential medicine policy**		.006	.002	.147	2.590	**.010** *
**Acquisition of pharmaceutical knowledge**		−.005	.002	−.111	**−**2.004	**.046** *

* Significant.

Doctors under 45 years old prescribed fewer antibiotics than those over 45 years old; doctors with bachelor’s degree prescribed more antibiotics than those with master’s degree or above. The percentage of antibiotics used by surgeons and obstetricians/gynecologists were higher than those prescribed by pediatricians (Table 6). Table 7 indicates that doctors with a bachelor’s degree prescribed fewer injection drugs than those with master’s degree or above, and internists and surgeons used fewer injection drugs than pediatricians did. Furthermore, the percentage of drugs prescribed from the national essential drug list by doctors with associate degrees and bachelor’s degrees was higher than those by doctors with master’s degree or above. Internists and surgeons prescribed more essential medicines than pediatricians, and the percentage of essential medicines per prescription increased along with the doctors’ acquisition of pharmaceutical knowledge (Table 8).

**Table 6:** Results of multiple linear regression analysis influencing factors on average percentage of antibiotics usage.

		Unstandardized coefficients		Standardized coefficients	t	P.
	Variable	B	Std. Error	Beta		
	**(Constant)**	.257	.052		4.930	.000
**Age**	**≤35 years old**	−.127	.061	−.386	**−**2.089	**.037** *
	**36–45 years old**	−.119	.048	−.354	**−**2.463	**.014** *
**Gender**	**Female**	.015	.027	.046	.561	.575
**Final academic degree**	**Associate’s degree**	.042	.045	.079	.944	.346
	**Bachelor’s degree**	.076	.034	.180	2.198	.**029** *
**Work experience**	**<10 years**	.105	.066	.313	1.588	.113
	**10–14 years**	.053	.058	.127	.906	.365
	**15–19 years**	.058	.057	.128	1.023	.307
	**20–24 years**	.040	.046	.082	.869	.385
**Daily outpatient number**	**<15**	−.048	.031	−.141	**−**1.570	.117
	**15–50**	−.040	.027	−.123	**−**1.462	.145
**Specialty**	**Internist**	.022	.027	.064	.819	.413
	**Surgeon**	.066	.030	.162	2.181	**.030** *
	**Obstetrician& gynecologist**	.076	.031	.207	2.472	**.014** *

* Significant.

**Table 7:** Results of multiple linear regression analysis influencing factors on Average Percentage of Injection Usage.

		Unstandardized coefficients		Standardized coefficients	t	P.
	Variable	B	Std. Error	Beta		
	**(Constant)**	.252	.055		4.571	.000
**Age**	**≤35 years old**	.084	.064	.244	1.316	.189
	**36–45 years old**	.022	.051	.063	.435	.664
**Gender**	**Female**	.015	.029	.044	.530	.597
**Final academic degree**	**Associate’s degree**	−.033	.047	−.059	−.696	.487
	**Bachelor’s degree**	−.075	.036	−.171	**−**2.074	.**039** *
**Work experience**	**<10 years**	−.047	.070	−.132	−.666	.506
	**10–14 years**	−.005	.061	−.010	−.074	.941
	**15–19 years**	−.024	.060	−.050	−.394	.694
	**20–24 years**	−.022	.049	−.043	−.455	.649
**Daily outpatient number**	**<15**	.046	.032	.128	1.416	.158
	**15–50**	.018	.029	.051	.609	.543
**Specialty**	**Internist**	−.068	.028	−.190	**−**2.427	**.016** *
	**Surgeon**	−.093	.032	−.218	**−**2.918	**.004** *
	**Obstetrician& gynecologist**	.004	.033	.010	.117	.907

* Significant.

**Table 8:** Results of multiple linear regression analysis influencing factors on average percentage of drugs prescribed from national essential drug list.

		Unstandardized coefficients		Standardized coefficients	t	P.
	Variable	B	Std. Error	Beta		
	**(Constant)**	.158	.074		2.117	.035
**Age**	**≤35 years old**	.105	.076	.245	1.390	.166
	**36–45 years old**	.098	.060	.223	1.621	.106
**Gender**	**Female**	.025	.034	.058	.744	.458
**Final academic degree**	**Associate’s degree**	.160	.056	.229	2.844	**.005** *
	**Bachelor’s degree**	.120	.043	.218	2.798	**.005** *
**Work experience**	**<10 years**	−.126	.083	−.287	**−**1.521	.129
	**10–14 years**	−.055	.072	−.101	−.753	.452
	**15–19 years**	−.041	.071	−.070	−.587	.558
	**20–24 years**	−.008	.057	−.013	−.145	.885
**Specialty**	**Internist**	.110	.038	.248	2.875	**.004** *
	**Surgeon**	.108	.034	.252	3.145	**.002** *
	**Obstetrician& Gynecologist**	.038	.033	.086	1.153	.250
**Acquisition of pharmaceutical knowledge**		.054	.038	.101	1.426	.**155** *

* Significant.

## Discussion

A physician’s prescribing pattern is a result of a series of complex factors, including internal characteristics and motivations as well as external factors like social environment and patient requirements. So far, there is no standard for prescription quality indicators worldwide. The differences in the proportion of injections used in different countries are mainly because of the major health problems and causes of death in a specific population [8].

In this study, the average number of drugs prescribed was 3.52 in all 5099 prescriptions with a maximum of 10, which was higher than the relevant domestic research findings of Baotou, Inner Mongolia (2.7), Beijing (2.63), Guangdong (2.36) and Guangxi (1.95) Provinces [9]–[12]. Compared to other countries, the average number of prescription drugs in this study was markedly higher than the WHO’s recommended value of 1.3 to 2.0. The number of prescription drugs in this study is even higher than those of developing countries like Zimbabwe (1.3), Sudan (1.4) and Palestine (1.3) [7], [13]. This may be due to indefinite diagnoses and unreasonable demands by patients. The stimulation of personal economic interests from rebates of pharmaceutical companies to doctors may also account for this phenomenon. The risk of adverse interactions between drugs is raised by unreasonable combined medication, which thereby increases patients’ health risks.

In this study, the percentage of drugs prescribed with generic names was 96.12%, which was lower than the domestic research results from Baotou (99.3%), Guangdong (100%), and Guangxi (99.78%), and did not reach the WHO standard [9], [11], [12]. Compared to other studies, the indicator was significantly higher than in India (15.1%), Nigeria (49.3%), Sri Lanka (78%), Laos (78%) and other developing countries [14]–[16]. There is a general consensus among WHO and international society that using generic names is an effective way to reduce patient drug costs. China also makes it clear that all of the prescription drugs must be written in generic names. With regard to the proportion of generic names used in prescriptions, this study implied an optimistic situation in the county hospitals in China; however, it is still necessary to make further improvements.

The ratio of antibiotics used per prescription was 29.9%, which was close to the WHO’s standard of no higher than 30% [13]. It was lower than Nigeria (72.8%), Zimbabwe (58%), Laos (47%), and Sri Lanka (47%) but higher than in Yemen (24.6%) and Saudi Arabia (20%) [15]–[20]. In this study, the proportion of antibiotics used was lower than that of other developing countries, revealing the preliminary effectiveness of “the special rectification activities for the rational antibacterial agents use” sponsored by the Chinese Ministry of Health. However, the irrational use of antibiotics still remains and satisfactory results have not yet been achieved. Further, management and constraints by government and hospitals are still needed.

The proportion of injection drugs used per prescription in the study was 20.02%, which was much higher than the WHO standard (1%) but slightly lower than Hogerzeil’s findings in Uganda, Sudan and Nigeria (36%–48%). The excessive and unnecessary use of injection drugs is not only a waste of medical costs, medical staff, time and medical equipment but also increases the patient’s risk of infection by viruses, like hepatitis C and AIDS [21].

It was found that doctors under the age of 45 prescribed fewer antibiotics than those who were over 45 years old. This may be because of the Chinese grading administration model for prescription, which means that doctors with higher professional title have the right to prescribe more types of antibiotics than those with a lower professional title. However, Letizia *et al*. found that, in the Lazio district of Italy, the younger physicians were likely to prescribe more medicines because they hoped to cover the patients’ needs [22]. This study also showed that doctors’ final educational degree was related to all of the prescription quality indicators. Doctors with higher degrees tended to prescribe more drugs and fewer essential medicines. Compared to doctors with master’s degree, doctors with associate’s degrees prescribed fewer generic drugs, and doctors with bachelor’s degrees prescribed more antibiotics and fewer injection drugs. A slight improvement in the rational use of drugs could be precipitated by the stringent regulations on the use of antimicrobial drugs and the requirements of using generics, which was complied by doctors of all levels, especially those with higher degrees.

As a result of multifactor analysis, the average number of drugs per prescription of internists was less than that of pediatricians. Surgeons prescribed fewer generic drugs and fewer injection drugs but more antibiotics and essential medicines compared to pediatricians. It seems that doctors with different specialties have significant differences in their prescription quality. This finding was consistent with the studies from Vallano *et al.* [23], which demonstrated that the scores of prescription quality indicators were largely associated with their professional categories, and different specialties could cause differences in doctors’ prescription knowledge and attitude.

In the present study, we also found that with the increase of doctor’s education and training experience on rational drug use, and the increase of their pharmaceutical knowledge, the proportion of drugs prescribed with generic names by doctors showed a slight decrease. One possible reason is that impressive education and training for doctors by pharmaceutical companies made the doctors more likely to use brand names rather than generic names. Hogerzeil *et al.* has demonstrated that basic and post-basic medical education should include specific training in rational prescribing [24]. Moreover, Figueiras *et al*. found that passive education strategies may simply modify knowledge but have no effect on prescribing behavior [25]. Our results indicated that the more the doctor was acquainted with pharmaceutical expertise, the higher the proportion of essential medicines used in their prescriptions. Similarly, results from Yukun *et al.* and Dai Weihui *et al*. revealed that the doctor’s familiarity with drugs would have an influence on their prescription behavior [6], [26].

In this study, the gender variable, which was connected to the average number of drugs prescribed, generic names, injections and essential medicines prescribed in single factor analysis, showed no significant differences in the multifactor analysis. This finding is different from the results of Orzella *et al*., which demonstrated that female doctors preferred to prescribe more drugs than male doctors [22]. As influential variable in the single factor analysis, doctor’s work experience had no significant association with the usage of generic names, essential medicines, and any other prescription quality indicators in the multifactor analysis. However, study by Andersen *et al*. indicated that long-time work experience would actuate doctors to follow their habit, instead of opting for the best drugs [27].

Prescription quality was linked to job satisfaction in the study of Melville et al. [28]. In our present study, job satisfaction had no significant association with any of the five prescription quality indicators. One possible reason is selection bias of the doctors in completing the questionnaires. Moreover, none of the investigated hospitals linked the doctors’ rewards to their prescription quality, and thus diluted the relationship between job satisfaction and prescription quality.

The two factors, patients’ demographic characteristics (such as sex, age, and education) and economic conditions (such as income and medical insurance), which were extracted from the study by factor analysis, have no significant association with the five prescription quality indicators neither in the single factor analysis nor in the multifactor analysis. These may occur because the customers in county hospitals in this study are mainly peasants. No significant diversity exists in the peasant’s education and income level in China, especially within the same province. A majority of peasants’ healthcare costs are covered with the New Rural Cooperative Medical Scheme. Our results are different from the results of Vallano *et al*., which demonstrated that older patients received more drugs, a higher rate of generic drugs, and fewer antibiotics [23]. The probable reasons might be the difference in the spectrum of disease and the educational level of the patients from various countries.

In summary, differences in doctors’ final academic degree, specialty, acquisition of medical knowledge and attitude toward the national essential medicine policy led to different prescribing patterns. Prescription quality mainly depends on doctor’s choices, but its improvement is not only the business of doctors themselves. Promoting doctors’ medical knowledge, reinforcing the publicity of rational drug use, and initiating performance evaluation for doctors are effective ways to improve prescription quality in Chinese county hospitals.
